# *Clostridium difficile* in wild rodents and insectivores in the Netherlands

**DOI:** 10.1111/lam.13159

**Published:** 2019-05-02

**Authors:** I.M. Krijger, B.G. Meerburg, C. Harmanus, S.A. Burt

**Affiliations:** ^1^ Livestock Research Wageningen University & Research Wageningen The Netherlands; ^2^ Farm Technology Group Wageningen University Wageningen The Netherlands; ^3^ Dutch Pest and Wildlife Expertise Centre (KAD) Wageningen The Netherlands; ^4^ Leiden University Medical Centre Leiden The Netherlands; ^5^ Division of Environmental Epidemiology & Veterinary Public Health Institute for Risk Assessment Sciences Faculty of Veterinary Medicine Utrecht University Utrecht The Netherlands

**Keywords:** animal to human, *Clostridioides difficile*, farms, house mouse, *Mus musculus*, *Rattus rattus*, transmission, zoonotic pathogen

## Abstract

With wild rodents and insectivores being present around humans and their living, working and food production environments, it is important to gain knowledge of the zoonotic pathogens present in these animals. The enteropathogen *Clostridium difficile*, an opportunistic anaerobic bacteria, can be carried by both animals and humans, and is distributed globally. It is known that there is genetic overlap between human and animal sources of *C. difficile*. In this study, the aim was to assess the presence of *C. difficile* in rodents and insectivores trapped on and around pig and cattle farms in the Netherlands. In total 347 rodents and insectivores (10 different species) were trapped and 39·2% tested positive for presence of *C. difficile*. For all positive samples the ribotype (RT) was determined, and in total there were 13 different RTs found (in descending order of frequency: 057, 010, 029, 005, 073, 078, 015, 035, 454, 014, 058, 062, 087). Six of the RTs isolated from rodents and insectivores are known to be associated with human *C. difficile* infection; RT005, RT010, RT014, RT015, RT078 and RT087. The presence of rodents and insectivores in and around food production buildings (e.g. farms) could contribute to the spread of *C. difficile* in the human environment. In order to enable on‐farm management for pathogen control, it is essential to comprehend the role of wild rodents and insectivores that could potentially affect the ecology of disease agents on farms.

**Significance and Impact of the Study:**

This study shows that rodents and insectivores in and around food production buildings (e.g. farms) can carry *Clostridium difficile* ribotypes associated with human *C. difficile* infection (CDI). *C. difficile* spores in rodent and insectivore droppings are able to survive in the environment for prolonged periods, leading to host‐to‐host exposure and transmission. Therefore we can state that rodent and insectivore presence on farms is a risk for zoonotic pathogen transmission of *C. difficile*.

## Abstract 2

**Significance and Impact of the Study:** This study shows that rodents and insectivores in and around food production buildings (e.g. farms) can carry *Clostridium difficile* ribotypes associated with human *C. difficile* infection (CDI). *C. difficile* spores in rodent and insectivore droppings are able to survive in the environment for prolonged periods, leading to host‐to‐host exposure and transmission. Therefore we can state that rodent and insectivore presence on farms is a risk for zoonotic pathogen transmission of *C. difficile*.

## Introduction

The opportunistic anaerobic bacteria *Clostridium difficile* is an enteropathogen for both humans and animals that is distributed globally (Freeman *et al*. 2010). There are more than 800 ribotypes (RTs) of *C. difficile* known and this Gram‐positive bacteria can be found in the intestinal tract of many animal species, but also in water, soil and on meat (Al Saif and Brazier 1996; Songer *et al*. 2009; de Boer *et al*. 2011; W. Fawley, personal communication). *C. difficile* infection (CDI) is one of the most frequently observed sources of mucosal injury and inflammation in hospital patients, leading to diarrhoea or inflammation of the colon (Kelly and LaMont 1998). However, it is also described in patients who did not visit the hospital (Chernak *et al*. 2005). CDI is an emerging disease, both in human patients and in animals used for food (Keessen *et al*. 2011; Balsells *et al*. 2018; Crobach *et al*. 2018; Rodriguez Diaz *et al*. 2018). The bacterium *C. difficile* not only causes disease in humans, it is also able to cause enteric disease in several animal species, such as horses, piglets, calves and other domestic animals (Båverud 2002; Rupnik 2007; Rupnik *et al*. 2009; Kecerova *et al*. 2019). This finding suggests that animals and humans may share a common source (Rupnik 2007), and it has been shown that there is substantial overlap of *C. difficile* strains present in humans and animals (Keessen *et al*. 2011; Rodriguez Diaz *et al*. 2018). This overlap of *C. difficile* types could indicate zoonotic spread amongst animals and humans. With wild rodents being present around humans and their living, working and food production environments, it is important to gain knowledge of the zoonotic pathogens present in these commensal rodents (Meerburg *et al*. 2009; Meerburg 2010; Himsworth *et al*. 2014) and insectivores. There are few studies published on the presence of *C. difficile* in rodents (Burt *et al*. 2012, 2018; Himsworth *et al*. 2014; Andrés‐Lasheras *et al*. 2017; de Oliveira *et al*. 2018) and even fewer in insectivores (Jardine *et al*. 2013). Therefore the aim of this study was to assess the presence of *C. difficile* in rodents and insectivores trapped on and around pig and cattle farms in the Netherlands. *C. difficile* spores in rodent droppings are able to survive in the environment for prolonged periods, which leads to numerous options for host‐to‐host exposure and transmission (Leffler and Lamont 2015; Knetsch *et al*. 2018). In order to enable pathogen control on farms, it is essential to understand the role of wild rodents and insectivores that could potentially affect the ecology of disease agents on farms (Rothenburger *et al*. 2018).

## Results and discussion

In total 347 rodents and insectivores were trapped with snap‐traps and tested for the presence of *C. difficile* (Table 1). Ten different species were analysed, three of which were insectivores; the greater white‐toothed shrew (*Crocidura russula*), the common shrew (*Sorex araneus*) and the crowned shrew (*Sorex coronatus*). Rodents were caught in greater numbers than insectivores, with the black rat (*Rattus rattus*) being predominant (53·6%), followed by the house mouse (*Mus musculus*, 24·2%). It was found that 39·2% (*n* = 347) of the trapped rodents tested positive for *C. difficile*. This percentage is in line with a previous study on *C. difficile* in rodents from the Netherlands, in which 35% of the rodents were positive (Burt *et al*. 2018). Similar to other previous studies on *C. difficile* in rodents (Himsworth *et al*. 2014; Burt *et al*. 2018), there was no association between gender and occurrence of *C. difficile* in the present work. This is in contrast to many other pathogens, for which male rodents have been shown to be more prone to infection (Meerburg *et al*. 2009).

**Table 1:** Overview of results of *Clostridium difficile* analysis per rodent and insectivore species and gender

Species	Type	Number of animals (no. positive for *C. difficile* between brackets)			
		Female	Male	Total	%
Wood mouse (*Apodemus sylvaticus*)	Rodent	10	9 (1)	19 (1)	5·3
Greater white‐toothed shrew (*Crocidura russula*)	Insectivore	1	1	2	0
Eurasian harvest mouse (*Micromys minutus*)	Rodent	1	0	1	0
Common vole (*Microtus arvalis*)	Rodent	4	4 (1)	8 (1)	12·5
House mouse (*Mus musculus*)	Rodent	36 (17)	48 (13)	84 (30)	35·7
Muskrat (*Ondatra zibethicus*)	Rodent	0	1 (1)	1 (1)	100
Brown rat (*Rattus norvegicus*)	Rodent	18 (3)	18	36 (3)	8·3
Black rat (*Rattus rattus*)	Rodent	100 (56)	86 (43)	186 (99)	53·2
Common shrew (*Sorex araneus*)	Insectivore	3	6 (1)	9 (1)	11·1
Crowned shrew (*Sorex coronatus*)	Insectivore	0	1	1	0
Total		173 (76)	174 (59)	347 (136)	39·2

The RT for all samples of rodent and insectivore intestinal content was determined, and 13 different RTs in total were found (in descending order of frequency: 057, 010, 029, 005, 073, 078, 015, 035, 454, 014, 058, 062, 087, Table 2). The black rat (*R. rattus*) and house mouse (*M. musculus*) are species with the highest diversity in RTs, 8 and 7 types respectively. The RT most frequently isolated was RT057, which was only found in black rats and house mice. Although present at such high percentages, no references to RT057 could be found in the literature. However, RT057 is also frequently found in humans and characterized as producing toxin A and B (unpublished data of the Dutch National Reference Laboratory for CDI). The fact that no literature was found on this RT could be due to the possibility that RT057 does not result in clinical symptoms in humans.

**Table 2:** *Clostridium difficile* ribotypes (RTs) confirmed in samples of the intestinal contents of wild rodents and insectivores in the Netherlands

RT	No. of isolates	Species
005^a^	10	*Mus musculus*,* Rattus rattus*,* Sorex araneus*
010^a^	12	*R. rattus*
014^a^	1	*R. rattus*
015^a^	2	*M. musculus*,* R. norvegicus*
029	12	*Apodemus sylvaticus*,* Microtus arvalis*,* M. musculus*
035	2	*M. musculus*
057	81	*M. musculus*,* R. rattus*
058	1	*R. rattus*
062	1	*R. rattus*
073	6	*M. musculus*
078^a^	5	*M. musculus*,* Ondatra zibethicus*,* R. rattus*
087^a^	1	*R. norvegicus*
454	2	*R. rattus*

a RT associated with *C. difficile* infection in humans.

Three insectivore species were tested, of which one (*S. araneus*) was found to carry *C. difficile* (RT005). Unfortunately, literature on *C. difficile* in shrews or other insectivores such as moles or hedgehogs is scarce. Only one published report could be found: a study in Canada assessed *C. difficile* in wild mammals, including two short‐tailed shrews (*Blarina brevicauda*) from around a dairy farm, one of which was found positive for *C. difficile* (Jardine *et al*. 2013).

It is known that there is genetic overlap between human and animal sources of *C. difficile* (Knight and Riley 2016; Crobach *et al*. 2018; Rodriguez Diaz *et al*. 2018). In this study, 6 RTs that are known to be associated with human CDI were isolated from rodents; RT005, RT010, RT014, RT015, RT078 and RT087. Below, we describe the four which were found in more than one of our samples.

In Europe, RT005 is a source of CDI in humans (Reil *et al*. 2012; Freeman *et al*. 2015) and is also associated with rodents. In a recent study from New York, RT005 was isolated from *M. musculus* (Williams *et al*. 2018). RT005 has also been described in pest species around pig farms (*M. musculus*,* Rattus* sp.) in Spain (Andrés‐Lasheras *et al*. 2017), in a Norway rat (*Rattus norvegicus*) in Canada (Himsworth *et al*. 2014) and in an urban mouse in the Netherlands (Burt *et al*. 2018).

In Europe, RT014 has also been found to cause CDI in humans (Freeman *et al*. 2015), and occurs prominently in Dutch CDI patients (Hensgens *et al*. 2009; Bauer *et al*. 2011) as well as in other European countries (Arvand *et al*. 2014; Indra *et al*. 2015). Of the isolated RT types, RT 014 occurs as most often reported type in the database of Dutch National Reference Laboratory for CDI since 2006 (see Table S1). RT014 is commonly found in pigs (Knight *et al*. 2015; Knight and Riley 2016; Martin *et al*. 2016). In previous studies, RT 014 was found in rodents as well (Himsworth *et al*. 2014; Burt *et al*. 2018; de Oliveira *et al*. 2018). Cats and dogs have been found to carry RT014 (Andrés‐Lasheras *et al*. 2018; Rabold *et al*. 2018), which could be linked to the rodents; as cats commonly hunt small rodents, *C. difficile* can possibly be transferred from rodent to cat.

A third RT isolated from the rodents/insectivores, which is known to be associated with human CDI, is RT078. This is a known causative agent for human CDI in Europe (Goorhuis *et al*. 2008a; Hensgens *et al*. 2010) and the most common RT present in pigs, causing diarrhoea in these animals (Keel *et al*. 2007; Goorhuis *et al*. 2008b; Debast *et al*. 2009). RT078 is the third‐most frequently found PCR RT in Dutch hospitals and in hospitals in several other European countries (Hensgens *et al*. 2009; Bauer *et al*. 2011). A study from 2012 (Burt *et al*. 2012) showed that *M. musculus* from a pig farm and other pest species present on the farm (insects, birds, rodent droppings and bird droppings) carried RT078. In Spain, RT078 was also found in rodents (*Rattus* sp. and *M. musculus*) on pig farms (Andrés‐Lasheras *et al*. 2017).

Another well‐known human RT is RT010, which was recently also found in dogs (Álvarez‐Pérez *et al*. 2015; Rabold *et al*. 2018) and in rabbits (Drigo *et al*. 2015). The occurrence of this strain in animals and humans suggests at least a common source of infection.

Evidence for zoonotic transmission of *C. difficile* (strain RT078) has only recently been reported by Knetsch *et al*. (2014, 2018), and for strain RT014, evidence was found for zoonotic transmission between pigs and humans (Knight *et al*. 2017). This transmission potential between animals and humans leads to a zoonotic risk, not only between humans and farm animals, but also pets and humans, and (indirectly) rodents and humans. This study concludes that wild rodents and insectivores are a reservoir for several *C. difficile* RTs, some of which are associated with human CDI. The presence of rodents and insectivores in and around food production buildings (e.g. farms) could contribute to the spread of *C. difficile* in the human environment. An interesting question to address during future research is whether the RTs found in these small mammals are also present in the environment if rodents and insectivores are absent. If so, this could mean that small mammals acquire infection from the environment and are then able to distribute the pathogen further throughout their habitat.

## Materials and methods

Small mammal trapping was conducted from November 2016 until January 2017 on 10 conventional pig farms and one dairy farm in the Netherlands distributed over the country. Rodents and insectivores were trapped using snap‐traps as part of standard pest‐control activities (cadavers were otherwise destined for disposal). The period between capture and storage was kept as short as possible to prevent for overgrowth (max 24 h). Trapped animals were stored in separate bags at −18°C. All specimens were thawed at 4°C 24 h before dissection. During dissection at the Wageningen Bioveterinary Research Institute, each animal was identified to species level and sexed. Samples of 2–4 droppings were collected from the ileum of each animal. Samples were stored at −20°C until further analysis.

### Analysis and ribotyping of the samples

Analysis of the rodent gut content for *C. difficile* was conducted following the procedure of Hopman *et al*. (2011), except for two alterations; (i) *C. difficile* enrichment broth was used (CDEB, Mediaproducts, Groningen, the Netherlands) in the enrichment phase and (ii) samples were incubated for 7 days in CDEB before plating out on agar (selective agents in CDEB were moxalactam and norfloxacine). Samples were classed as positive for *C. difficile* if they produced colonies of Gram‐positive rods with a characteristic odour of horse manure and typical morphology (grey colonies with an uneven edge). Isolates were further identified and characterized at the National Reference Laboratory at Leiden, the Netherlands by capillary ribotyping (Bidet *et al*. 2000) following the consensus protocol as described by Fawley *et al*.(2015).

### Statistical analysis

The results of the *C. difficile* analysis were compared between the genders of the rodents and insectivores caught, using an independent samples *T*‐test using IBM spss statistics software, ver. 23 (IBM Corp., Armonk, NY).

## Conflict of Interest

No conflict of interest declared.

## Supporting information


**Table S1** Occurrence of the *Clostridium difficile* ribotypes from this study in the Dutch human database since 2006 (unpublished data of the Dutch National Reference Laboratory for *C. difficile* infections).
