# Supplementary material for: Clostridium difficile in wild rodents and insectivores in the Netherlands
Source: Lett Appl Microbiol. 2019 May 2;69(1):35–40. doi: 10.1111/lam.13159 (PMC6849583; doi:10.1111/lam.13159)
Supplement: Supplementary file 1 — Table S1 Occurrence of the Clostridium difficile ribotypes from this study in the Dutch human database since 2006 (unpublished data of the Dutch National Reference Laboratory for C. difficile infections). [file LAM-69-35-s001.docx]

**Supporting Information**

**Table S1**. Occurrence of the *Clostridium difficile* ribotypes from this study in the Dutch human database since 2006 (unpublished data of the Dutch National Reference Laboratory for *C. difficile* infections).

| Ribotype (RT) | Number of times reported in Dutch National Reference Laboratory |
| --- | --- |
| 005^●^ | 663 |
| 010^●^ | 116 |
| 014^●^ | 2291 |
| 015^●^ | 515 |
| 029 | 143 |
| 035 | 13 |
| 057 | 58 |
| 058 | 1 |
| 062 | 33 |
| 073 | 17 |
| 078^●^ | 1496 |
| 087^●^ | 180 |
| 454 | 11 |

^●^RT associated with CDI in humans

All RT types were isolated from human faeces sent to the Dutch National Reference Laboratory for *C. difficile* infections. The fact that an RT type is isolated does not necessarily mean that the person has CDI as we do not have insight in clinical information on the samples.
